# Task-specific training for improving propulsion symmetry and gait speed in people in the chronic phase after stroke: a proof-of-concept study

**DOI:** 10.1186/s12984-021-00858-8

**Published:** 2021-04-23

**Authors:** J. F. Alingh, B. E. Groen, J. F. Kamphuis, A. C. H. Geurts, V. Weerdesteyn

**Affiliations:** 1 Sint Maartenskliniek Research, PO Box 9011, 6500 GM Nijmegen, The Netherlands; 2 Department of Rehabilitation, Radboud University Medical Center, Donders Institute for Brain, Cognition and Behaviour, Nijmegen, The Netherlands; 3 Department of Rehabilitation, Sint Maartenskliniek, Nijmegen, The Netherlands

**Keywords:** Stroke, Rehabilitation, Gait, Robotics, Propulsion, Speed, Biomechanics

## Abstract

**Background:**

After stroke, some individuals have latent, propulsive capacity of the paretic leg, that can be elicited during task-specific gait training. The aim of this proof-of-concept study was to investigate the effect of five-week robotic gait training for improving propulsion symmetry by increasing paretic propulsion in chronic stroke survivors.

**Methods:**

Twenty-nine individuals with chronic stroke and impaired paretic propulsion (≥ 8% difference in paretic vs. non-paretic propulsive impulse) were enrolled. Participants received ten 60-min sessions of individual robotic gait training targeting paretic propulsion (five weeks, twice a week), complemented with home exercises (15 min/day) focusing on increasing strength and practicing learned strategies in daily life. Propulsion measures, gait kinematics and kinetics, self-selected gait speed, performance of functional gait tasks, and daily-life mobility and physical activity were assessed five weeks (T0) and one week (T1) before the start of intervention, and one week (T2) and five weeks (T3) after the intervention period.

**Results:**

Between T0 and T1, no significant differences in outcomes were observed, except for a marginal increase in gait speed (+ 2.9%). Following the intervention, propulsion symmetry (+ 7.9%) and paretic propulsive impulse had significantly improved (+ 8.1%), whereas non-paretic propulsive impulse remained unchanged. Larger gains in propulsion symmetry were associated with more asymmetrical propulsion at T0. In addition, following the intervention significantly greater paretic trailing limb angles (+ 6.6%) and ankle plantarflexion moments (+ 7.1%) were observed. Furthermore, gait speed (+ 7.2%), 6-Minute Walk Test (+ 6.4%), Functional Gait Assessment (+ 6.5%), and daily-life walking intensity (+ 6.9%) had increased following the intervention. At five-week follow-up (T3), gains in all outcomes were retained, and gait speed had further increased (+ 3.6%).

**Conclusions:**

The post-intervention gain in paretic propulsion did not only translate into improved propulsion symmetry and gait speed, but also pertained to performance of functional gait tasks and daily-life walking activity levels. These findings suggest that well-selected chronic stroke survivors may benefit from task-specific targeted training to utilize the residual propulsive capacity of the paretic leg. Future research is recommended to establish simple baseline measures for identification of individuals who may benefit from such training and confirm benefits of the used training concepts in a randomized controlled trial.

*Trial registration:* Registry number ClinicalTrials.gov (www.clinicaltrials.gov): NCT04650802, retrospectively registered 3 December 2020.

**Supplementary Information:**

The online version contains supplementary material available at 10.1186/s12984-021-00858-8.

## Introduction

While the majority of stroke survivors regain independent walking [1], gait efficiency and speed are often persistently reduced compared to healthy adults [2]. Post-stroke gait speed is associated with community ambulation, as a minimum speed of 0.4 m/s seems necessary for walking outside the home, and a speed faster than 0.8 m/s seems required for full community ambulation [3, 4]. In addition, impaired post-stroke gait speed is associated with reduced quality of life [5, 6]. Hence, a common goal for post-stroke rehabilitation interventions is to improve gait speed.

Gait speed is mainly generated by ankle push-off force during terminal stance, which helps propel the body forward. Gait propulsion is usually defined as the horizontal component of the ground reaction force during push-off. Propulsion is determined by the ankle plantarflexion moment [7], in combination with the angle of the trailing limb with the vertical during push-off [8–10]. Generally, larger trailing limb angles are associated with more anteriorly directed ground reaction forces [11], resulting in a larger contribution of the ankle plantarflexion moment to forward (instead of upward) acceleration of the body. After stroke, propulsion of the paretic leg is often lower than the values observed in healthy adults [12–14]. This is probably due to muscle weakness [9, 15, 16], loss of selective motor control [17], and/or balance uncertainty and reduced limb loading [18]. Reductions in paretic compared to non-paretic propulsion result in propulsion asymmetry [19], which is associated with impaired walking capacity [19–21]. In addition, deficits in paretic propulsion are associated with reduced paretic knee flexion during swing [22, 23], which may affect walking efficiency [24, 25] and increase the risk of falling [26]. In order to compensate for the lack of paretic propulsion, stroke survivors tend to rely more on the non-paretic leg’s propulsion generation [19, 27], as well as on paretic hip pull-off to progress the paretic leg during swing [14, 16]. These compensatory mechanism are, however, associated with reduced gait efficiency [25, 28]. Increasing the contribution of the paretic leg to propulsion is, therefore, a key target for restoring gait post stroke [29].

A recent review of studies evaluating propulsion and gait speed after single or multiple training sessions suggested that individuals in the chronic phase after stroke may not fully utilize their residual propulsive capacity, possibly due to ‘learned non-use’ of the paretic leg [30]. It was suggested that targeted and challenging training focusing on stronger ankle plantarflexion and larger trailing limb angle may help people with stroke reactivate this latent propulsive capacity of the paretic leg, thus improving propulsion symmetry [21, 30, 31]. Yet, to date only few studies involved training programs primarily aimed at improving propulsion in individuals in the chronic phase after stroke [32–37], of which some evaluated the long-term training effects [32–34]. Overall, these studies yielded mixed results [32–37]. Their findings suggest that the latent propulsive capacity of the paretic leg can be elicited during task-specific training in individuals with chronic stroke, but it remains questionable if benefits are retained over time.

The primary aim of this study was to investigate the effect of a five-week gait training for improving propulsion symmetry by increasing propulsion of the paretic leg in individuals in the chronic phase after stroke. The training was conducted in robotic gait trainer LOPES II [38]. LOPES II training allowed participants to focus attention on their paretic leg, attributable to the provided balance support and guided weight shifts. Compensatory movements could be reduced through mechanical assistance of the lower limbs (by LOPES II) and by providing real-time feedback of the individual’s gait performance. Propulsion was challenged by increasing step length and velocity, or moving against robotic resistance. In addition to paretic leg propulsion, we also determined its constituent factors, namely the trailing limb angle and the ankle plantarflexion moment of the paretic leg. Our secondary aim was to determine whether the capacity of participants to increase their paretic propulsive impulse at baseline would be an indicator of the latent propulsive capacity of the paretic leg [39] and, thus, a relevant patient-related predictor of a positive training response. In addition, we assessed paretic knee flexion during swing (ICF-impairment level); self-selected gait speed and functional gait tasks (ICF-capacity level); and daily-life mobility impact and physical activity (ICF-performance level). We hypothesized that five weeks (ten sessions) of gait training in LOPES II would improve propulsion symmetry and, thereby, gait speed and execution of functional gait tasks. In addition, we expected that improved gait capacity might lead to a lower impact of stroke on daily-life mobility and a higher physical activity level.

## Methods

### Participants

Individuals in the chronic phase after stroke were recruited between December 2018 and December 2019 from the outpatient departments of the Radboud University Medical Center and the Sint Maartenskliniek (Nijmegen, the Netherlands). Inclusion criteria were: (1) adult age (≥ 18 years), (2) unilateral, ischemic or hemorrhagic, supratentorial stroke longer than 6 months post onset, (3) impaired propulsion of the paretic leg during walking at self-selected speed (i.e. ≥ 8% difference in paretic vs non-paretic propulsive impulse), (4) capacity to walk 10 m without support or use of a walking aid (Functional Ambulatory Categories/FAC 3–5 [40]), and (5) capacity to walk for five consecutive minutes, with or without the use of a walking aid. Exclusion criteria were: (1) inability to move the body upward against gravity while standing on both legs (loss of calf muscle strength assessed with the Medical Research Council/MRC scale < 3 [41]), (2) severe cognitive problems assessed with the Mini-Mental State Examination (MMSE < 24 [42]), (3) depressed mood assessed with the Hospital Anxiety and Depression Score (HADS > 7 [43]), (4) persistent unilateral visuospatial neglect assessed with the Star Cancellation Test (score < 44 [44]), (5) any medical condition interfering with gait, (6) inability to understand verbal instructions, or (7) inappropriate or unsafe fitting of the robotic gait trainer, due to severe lower limb spasticity (Modified Ashworth Scale (MAS) ≥ 3 [45]), severe lower limb contractures, body weight ≥ 140 kg, or skin problems at body sites where the harness or straps were to be fitted. After inclusion, the following demographic and clinical characteristics were collected: sex, age (years), type of stroke (ischemic/hemorrhagic), time since stroke (months), hemiparetic side, ambulatory capacity (FAC; range 0–5), lower limb motor selectivity (Fugl Meyer Assessement—leg score 0–34 [46]), lower limb strength (Motricity Index—leg score 0–100 [47]). The study protocol (NL 62617.091.17) was approved by the Medical Ethical Board of the region Arnhem-Nijmegen (The Netherlands). All procedures were conducted in accordance with the Declaration of Helsinki. Written informed consent was obtained for all participants.

### Study design

We conducted a longitudinal intervention study with two consecutive baseline assessments and a five-week follow-up to determine proof of concept. Assessments were performed five weeks (T0) and one week (T1) before the start of the intervention, and one week (T2) and five weeks (T3) after the end of the five-week intervention period.

### Intervention

Each participant received two 60-min sessions of individual robotic gait training per week, for five weeks, to target paretic propulsion. Robotic gait training was performed using LOPES II, a treadmill based exoskeleton, combined with a body-weight support system (MOOG BV, Nieuw-Vennep, the Netherlands). For a detailed description of the LOPES II see Meuleman et al. [38]. All training sessions were delivered by an experienced LOPES II trainer. To help elicit the latent propulsive capacity of the paretic leg, the robotic gait training included three key elements. First, weight shift guidance was applied to the pelvis and levels of body-weight support were set to a minimum, to improve weight acceptance on the paretic leg, necessary for push-off [18]. Second, minimal levels of general guidance force were applied to help participants match their gait pattern with the reference trajectory of the LOPES II, thereby reducing compensatory movements that may limit the need to generate paretic propulsion. If tolerated, the robotic guidance force was gradually reduced over time, while striving for a normal gait pattern. Third, step length and gait speed were increased and, if possible, participants were asked to move against the robotic assistance, to even further challenge the propulsion of the paretic leg. Across training sessions, progressive training intensity was provided by increasing gait speed, reducing assistance and limiting resting breaks. During each training session, participants received real-time feedback of the targeted gait parameter (i.e., weight shift, hip extension, estimated push-off, or step length) by the user interface of the LOPES II, which was projected on a tv-screen in front of the participant. Additionally, participants received verbal feedback from the LOPES II trainer. Training settings were recorded in a logbook. The robotic gait training was complemented with daily, 15-min home exercises. The home exercises consisted of two parts. The first part contained exercises to bilaterally improve calf muscle strength (e.g. standing heel raises, and forward or backward step-up). The second part consisted of exercises to practice the learned strategies to increase paretic propulsion in daily life (e.g. weight acceptance on the paretic leg in stance and during stepping, and level walking with variable speed or step length). The frequency and duration of the performed home exercises were recorded in a logbook.

### Outcome measures

At each assessment a 3D gait analysis and functional gait tasks were performed. In addition, daily-life mobility and physical activity were evaluated. For the 3D gait analysis, reflective markers (n = 39) were attached to the body according to the Plug-In-Gait Full Body model (Plug-In-Gait, Vicon Motion Systems, Ltd, Oxford, UK), and recorded by eight infrared cameras (f_s_ = 100 Hz; Vicon mX 1.7.1, Oxford Metrics, UK). Participants wore their own shoes. Use of a walking aid or ankle–foot orthosis was not allowed. Participants were instructed to walk at their self-selected, comfortable speed along a straight six-meter walkway with two embedded force plates (Kistler, Kistler Group, Winterthur, Switzerland) to record 3D ground reaction force data (f_s_ = 1000 Hz). At least five strides were collected in which either of both feet hit the respective force plate. During the 3D gait analysis at T0, participants were also asked to walk along the walkway at a fast speed, during which at least five strides were collected where both feet hit the respective force plates. Functional gait tasks included the 6-Minute Walk Test (6MWT [48]) and the Functional Gait Assessment (FGA; range 0–30 [49]). Daily-life mobility and physical activity were assessed with the Stroke Impact Scale (SIS—domain Mobility; range 0–100 [50]) and an activity tracker (Activ8, Remedy Distribution Ltd., Valkenswaard, The Netherlands), respectively. The activity tracker Activ8 has been shown to be sufficiently accurate in detecting daily-life physical activity in individuals after stroke [51]. At the end of each assessment, the activity tracker was attached to the non-paretic thigh using waterproof skin tape. The week following each assessment, participants wore the activity tracker for 24 h a day, for a minimum of five consecutive days.

### Data analysis

Custom written software (MATLAB, Mathworks Inc, Natrick, MA, USA) was used to analyze the data of the 3D gait analysis. Ground reaction force data were filtered with a low-pass, fourth order, bidirectional, Butterworth filter at 10 Hz. The primary outcome was propulsion symmetry at self-selected speed. For each trial, we calculated the propulsive impulse of the paretic and the non-paretic leg as the time integral of the anterior ground reaction force during the stance phase of gait, normalized for the individual’s body weight (N/s/kg). Propulsion symmetry was calculated by dividing the paretic propulsive impulse by the sum of the paretic and non-paretic propulsive impulses [19]. Self-selected gait speed (m/s) and paretic leg trailing limb angle (° [11]) were determined for each stride collected during the 3D gait analysis, using the position data of the C7 marker, and the position of the hip joint center and toe marker, respectively. In addition, Vicon Plug-In-Gait model and software were used to calculate paretic ankle plantarflexion moment (Nm/kg) for each stride. The trailing limb angle and ankle plantarflexion moment of the paretic leg were calculated at the instant of peak paretic anterior ground reaction force. Vicon Plug-In-Gait model and software were also used to determine peak paretic knee flexion during swing. At T0, a ‘propulsion capacity score’ was determined, which was defined as the difference in paretic propulsive impulse during walking at fast vs. self-selected speed of the gait strides obtained during the 3D gait analysis. The propulsion capacity score was used to determine the association between baseline capacity to increase paretic propulsive impulse and the training response. Mobility data of the activity trackers was analyzed using Activ8 software. Total time (minutes/day) and intensity (counts/minute) of walking were determined per day, and averaged over the number of days (minimum of five days) that the activity tracker was worn per assessment.

### Power calculation

Power analysis performed using STATA version 13 revealed that a sample size of 21 participants (α = 0.05; β = 0.20) was sufficient to show a difference in propulsion symmetry of 2.73 ± 4.32% (half the intervention effect reported by Awad et al. [32]) after the intervention. To determine the association between two relevant patient-related factors and a positive response to training, considering a rule of thumb to include 10–15 participants per predictor and taking into account a drop-out rate of 10%, we aimed for inclusion of 33 participants.

### Statistical analysis

Statistical analyses were performed using SPSS statistics version 25 (IBM Statistics, Chicago, USA). Propulsion symmetry, self-selected gait speed, paretic trailing limb angle, and paretic ankle plantarflexion moment were averaged per individual across all strides per assessment (T0-T3). Changes in baseline values between T0 and T1 were determined for each outcome measure using a paired-samples *t*-test or Wilcoxon Signed Rank Test, depending on data distribution. To assess changes in propulsion symmetry, propulsion impulse of the paretic and non-paretic leg, paretic ankle plantarflexion moment, paretic trailing limb angle, self-selected gait speed, performance on the 6MWT and FGA, and daily-life mobility and physical activity, linear mixed models for repeated measures were fit. The linear mixed models included as fixed effects: (1) the main effect of intervention (‘Intervention effect’, combined score of T0 and T1 vs. combined score of T2 and T3), (2) a covariate ‘baseline value’ at T0 (‘Baseline’), (3) an interaction effect of baseline with intervention effect (‘Intervention*Baseline interaction’), and (4) the effect of follow-up (‘Follow-up effect’, T2 vs. T3). In addition, the effect of intervention on peak paretic knee flexion during swing was analyzed for a subgroup of participants with reduced peak knee flexion at T0 (peak knee flexion ≤ 54° [52]). Since no changes were found between peak paretic knee flexion at T0 and T1 in this subgroup, peak paretic knee flexion at T0 was used as a reference and compared to peak paretic knee flexion at T2, using a Wilcoxon Signed Rank tests. Furthermore, to determine whether the propulsion capacity score at T0 was associated with the effect of intervention on propulsion symmetry (T0 vs. T2), a linear mixed model was fit which included as fixed effects: (1) the propulsion capacity score at T0, and (2) a covariate ‘propulsion symmetry at T0′. Results of the mixed models were obtained using a restricted maximum likelihood estimation, and an autoregression variance–covariance matrix to account for the correlation between the repeated measures (if applicable). The significance level was set at p ≤ 0.05 for all tests.

## Results

Twenty-nine individuals in the chronic phase after stroke were included in this study. Table 1 provides an overview of the baseline characteristics of the participants. The participants completed a median of 9.1 robotic gait training sessions (range: 7–10 training sessions). In addition, they completed a median of 21 (range: 15–33) sessions of home exercises. Due to technical problems, the 3D gait analysis at T1 could not be performed in one participant. Moreover, the follow-up assessment (T3) of six participants could not be performed due to lab closure as a result of the COVID-19 pandemic. As such, data of 23 participants was analyzed at T3. No adverse events were reported.

**Table 1:** Baseline demographic and clinical characteristics (mean ± SD or number) of the participants (N = 29)

Sex, male/female (n)	12 / 17
Age (years)	61.0 ± 8.1
Type of stroke, ischemic/hemorrhagic (n)	25 / 4
Time since stroke (months)	21.2 ± 10.7
Hemiparetic side, left/right (n)	15 / 14
FAC (n)	
3	9
4	16
5	4
Self-selected walking speed (m/s)	1.03 ± 0.21
Fugl-Meyer Assessment—leg score (0–34)	23.6 ± 4.9
Motricity index—leg score (0–100)	72.8 ± 9.0%
MRC—calf muscle (n) (0–5)	
3	16
4	8
5	5
MMSE (0–30)	28.2 ± 2.5
HADS—depression (0–21)	2.7 ± 2.4
Star Cancellation Test (0–54)	51.5 ± 2.9

*FAC score* Functional Ambulatory Categories, *MRC* Medical Research Council scale, *MMSE* Mini-Mental State Examination, *HADS* Hospital Anxiety and Depression Scale - subscale depression

### Propulsion measures

**Fig. 1:**
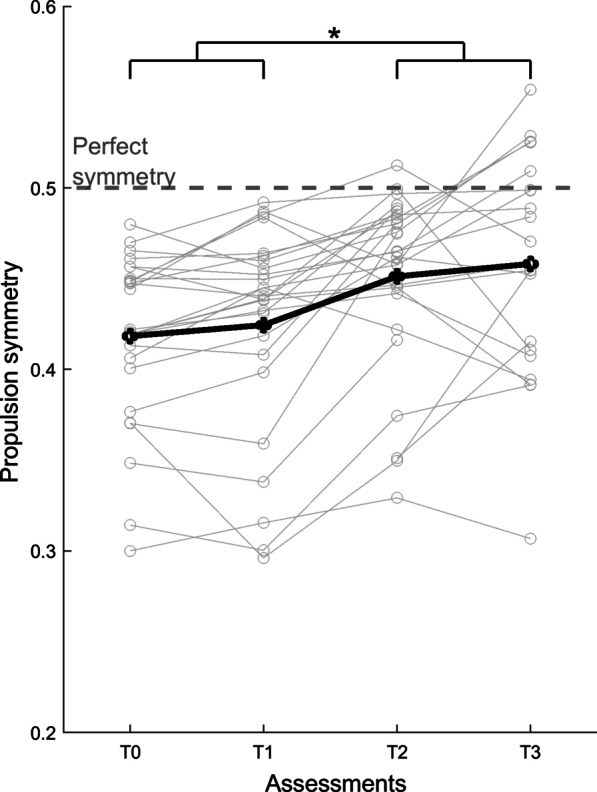
Average group (black line) and individual (grey lines) propulsion symmetry scores across assessments (T0-T3). A value of 0.5 represents perfect symmetry. *Significant difference between baseline (combined scores of T0 and T1) and post-intervention (combined scores of T2 and T3), p < 0.05

Between T0 and T1, mean propulsion symmetry, and paretic and non-paretic propulsive impulse did not significantly differ (p ≥ 0.114). Figure 1 shows propulsion symmetry over time. The corresponding test statistics of the mixed models are reported in Additional file 1. Following the intervention, mean propulsion symmetry had significantly improved by 7.9% (see Table 2; *Intervention* effect, p < 0.001), whereas it did not differ between post-intervention and follow-up (*Follow-up* effect, p = 0.083). Greater improvements in propulsion symmetry were observed in participants with more asymmetric values at baseline (Intervention*Baseline interaction, p < 0.001). The gain in propulsion symmetry was not associated with the propulsion capacity score at T0 (mean ± SD: 0.03 ± 0.03 N/s/kg; p = 0.984).

**Table 2:** Means (± SDs) of propulsion measures, capacity measures, and daily-life mobility and physical activity assessed 5 weeks before (T0), 1 week before (T1), immediately after (T2) and 5 weeks after (T3) the intervention period

	T0 n = 29	T1 n = 28^e^	T2 n = 29	T3 n = 23
*Propulsion measures*				
Propulsive impulse				
Symmetry^a,b^	0.42 ± 0.04	0.42 ± 0.05	0.45 ± 0.05	0.46 ± 0.06
Paretic leg (N/s/kg)^a^	0.21 ± 0.07	0.22 ± 0.08	0.23 ± 0.07	0.24 ± 0.08
Non-paretic leg (N/s/kg)	0.27 ± 0.06	0.28 ± 0.06	0.26 ± 0.08	0.27 ± 0.08
Trailing limb angle—paretic leg (°)^a,b^	11.7 ± 4.8	12.8 ± 5.1	12.9 ± 4.3	13.3 ± 4.7
Ankle plantarflexion moment—paretic leg (Nm/kg)^a,b^	12.1 ± 3.5	11.8 ± 3.9	12.9 ± 3.8	12.7 ± 3.1
*Capacity measures*				
Gait speed (m/s) ^a,b,c,d^	1.04 ± 0.20	1.07 ± 0.22	1.11 ± 0.21	1.15 ± 0.19
6MWT (m)^a^	429.5 ± 116.7	434.0 ± 117.7	456.3 ± 112.6	463.4 ± 124.5
FGA (0–30)^a^	19.0 ± 3.0	19.0 ± 2.6	20.3 ± 2.7	20.2 ± 2.7
*Daily-life mobility and physical activity*				
SIS—Mobility (0–80)	48.8 ± 3.4	49.4 ± 4.0	52.6 ± 4.5	51.7 ± 4.2
Activ8 walking				
Total time (min/day)	112 ± 40	108 ± 41	113 ± 40	115 ± 40
Total intensity (counts/day)^a^	1198 ± 306	1174 ± 306	1241 ± 286	1300 ± 310

*6MWT* 6-Minute Walk Test, *FGA* Functional Gait Assessment, *SIS* Stroke Impact Scale

^a^Significant difference between baseline (combined scores of T0 and T1) and post-intervention (combined scores of T2 and T3), p ≤ 0.05

^b^Significant *Intervention* * *Baseline* interaction, p ≤ 0.05

^c^Significant difference between T0 and T1, p ≤ 0.05

^d^Significant difference between T2 and T3, p ≤ 0.05

^e^Propulsion measures and gait speed are reported for 28 participants, whereas all other outcomes evaluated at T1 are reported for 29 participants

Following the intervention, the change in propulsion symmetry was accompanied by a significant increase in mean paretic propulsive impulse (8.1%; *Intervention* effect, p = 0.032), whereas no significant change of the mean non-paretic propulsive impulse was observed (*Intervention* effect, p = 0.190). During follow-up, neither paretic nor non-paretic propulsive impulse showed any change (*Follow-up* effect, p ≥ 0.724).The gain in paretic propulsive impulse following the intervention was not associated with the paretic propulsive impulse at T0 (Intervention*Baseline interaction, p = 0.183).

The mean trailing limb angle and mean ankle plantarflexion moment of the paretic leg did not differ between T0 and T1 (p ≥ 0.421). Following the intervention, these variables had significantly increased by 6.6% and 7.1%, respectively (*Intervention* effect, p ≤ 0.002), but did not change between post-intervention and follow-up (*Follow-up* effect, p ≥ 0.291). Greater improvements in trailing limb angle and ankle plantarflexion moment were observed in participants with smaller baseline trailing limb angle and ankle plantarflexion moment, respectively (Intervention*Baseline interaction, p ≤ 0.008).

### Capacity measures

**Fig. 2:**
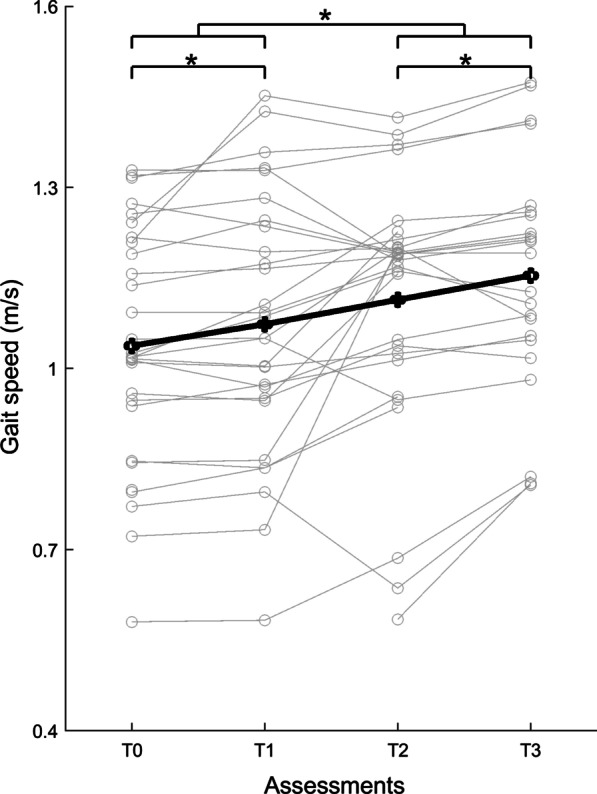
Average group (black line) and individual (grey lines) gait speed across assessments (T0-T3). *Significant differences between assessments T0 and T1, between assessments T2 and T3, and between baseline (combined scores of T0 and T1) and post-intervention (combined scores of T2 and T3), p < 0.05

Mean self-selected gait speed had significantly increased by 2.9% between T0 and T1 (*t*(27) = 2.146, p = 0.042), and showed a significant further increase of 7.2% following the intervention (*Intervention* effect, p < 0.001), and another 3.6% increase between post-intervention and follow-up (*Follow-up* effect, p = 0.050; see Fig. 2). Greater increases in gait speed were observed in participants with a slower gait speed at baseline (Intervention*Baseline interaction, p < 0.001). Mean scores on the 6MWT and FGA did not significantly differ between T0 and T1 (p ≥ 0.327), significantly improved following the intervention by 6.4% and 6.5%, respectively (*Intervention *effect, p < 0.019), but did not change between post-intervention and follow-up (*Follow-up* effect, p ≥ 0.175). The gain in performance on the 6MWT and FGA was not associated with baseline scores at T0 (Intervention*Baseline interaction, p ≥ 0.148).

### Daily-life mobility and physical activity

Mean scores on the SIS-Mobility and the mean total walking time per day did not significantly differ between T0 and T1 (p ≥ 0.202), nor following the intervention (*Intervention* effect, p ≥ 0.108), or during follow-up (*Follow-up* effect, p ≥ 0.122). The total intensity of walking did not differ between T0 and T1 (p = 0.248), significantly increased by 6.9% following the intervention (*Intervention* effect, p = 0.003), but did not change during follow-up (*Follow-up* effect, p = 0.496). The increase in total intensity of walking following the intervention was not associated with the intensity of walking at T0 (Intervention*Baseline interaction, p = 0.056).

### Knee flexion during swing

For participants with reduced peak paretic knee flexion during swing at T0 (N = 9, mean ± SD: 36.4 ± 13.9°), peak paretic knee flexion during swing had increased at T2 (mean ± SD: 46.4 ± 12.4°), which difference bordered significance (p = 0.051). Peak knee flexion data at T3 were only available for seven participants (mean ± SD: 46.5 ± 16.5°). This sub-group was considered too small to allow further statistical analysis.

## Discussion

Individuals in the chronic phase after stroke received five weeks of training targeting propulsion generation during gait. In line with our hypothesis, we found that propulsion symmetry had improved following the intervention, which improvement could be attributed to a larger contribution of the paretic leg. Propulsion generated by the non-paretic leg remained constant over time. The increase in paretic propulsion was observed in parallel with greater paretic ankle plantarflexion moments as well as larger paretic trailing limb angles. Individuals with more asymmetrical propulsion at baseline showed larger gains in propulsion symmetry following the intervention, whereas the ability to increase paretic propulsion during walking at a faster speed at baseline (propulsion capacity score) was not correlated with the intervention effect. Following the intervention, self-selected gait speed, performance on the 6-Minute Walk Test and the Functional Gait Assessment, and the intensity of walking in daily life had also increased. At five-week follow-up, the gains in all of these outcome measures were retained.

Our findings strongly support the emerging notion that in the chronic phase after stroke, paretic propulsion can be improved by targeted interventions [30]. The observation that paretic propulsion and gait speed improved concurrently is in line with previous studies [32, 34, 36, 37, 53]. Our results also confirmed previous reports of retention of these concurrent improvements during follow-up periods from six weeks to six months [32, 34, 53]. Notably, our results were obtained after only ten task-specific training sessions in five weeks, whereas previous task-specific training included six to 12-weeks of training, three times a week [32, 34, 36, 37, 53]. As propulsion is a key determinant of gait speed, it is likely that the increase in gait speed can be attributed to improvements in propulsion. Contradictory to our findings, some previous studies reported gains in gait speed without changes in paretic propulsion following gait interventions [33, 54, 55]. An increase in gait speed in the absence of improvements in paretic propulsion points at the use of compensatory mechanisms to overcome the lack of paretic propulsion. For example, Combs et al. [33] reported a greater contribution of the non-paretic, instead of the paretic leg to propulsion generation when stroke survivors increased their gait speed following training. Interestingly, most studies demonstrating gains in speed without changes in paretic propulsion did not specifically focus on propulsion. The primary outcomes of these studies were related to independent walking capacity [55] or gait performance [54]. In contrast, four out of five studies that did report concurrent improvements in speed and propulsion specified both primary aim and primary outcome at the level of paretic propulsion [32, 34, 36, 37]. These findings suggest that improvements in paretic propulsion do not merely emerge as a by-product of generic gait training, but require intervention strategies that specifically focus on this particular aspect of gait.

The improvement in paretic propulsive impulse was caused by an increase in both constituents of propulsion: the trailing limb angle [8–10] and the ankle plantarflexion moment [7, 8]. Larger trailing limb angles yield a better biomechanical position for propulsion generation by the ankle muscles [9], as the ground reaction force is directed more anteriorly [11]. In the current training, we applied several methods aimed at increasing the trailing limb angle. When participants walked with reduced hip extension, guidance force was applied to help them match the hip extension reference trajectory of the robot. In addition, participants were encouraged to increase their paretic trailing limb angle by walking with increased step length or gait speed. Apart from the trailing limb angle, ankle plantarflexion moment also increased. In the current training, the use of ankle plantarflexion capacity was challenged by increasing gait speed and by imposing a robotic resistance that the participants had to move against. In addition to the supervised training, part of the exercises that participants performed at home were focused on bilaterally improving calf muscle strength. It therefore remains unclear whether the observed increase in ankle moment was due to the task-specific training in the robotic gait trainer, the muscle strengthening exercises, or a combination of both. Yet, previous studies that involved strength training did not find differences in post-stroke ankle kinetics [14, 56]. We therefore consider the task-specific gait training to have been a key contributor to the observed increase in ankle plantarflexion moments.

The improvements in paretic propulsion are presumably unrelated to ‘true’ neurobiological recovery, as restitution of function is not expected to occur in the chronic phase [57]. In this phase, improvements in motor performance often result from learning new strategies to compensate for the existing impairments of the paretic limb [58]. For instance, stroke survivors may exaggerate propulsion of the non-paretic leg during gait [12, 19]. Yet, it is difficult to reconcile the observed improvements in paretic propulsion with a compensatory mechanism. Here, remission of ‘learned non-use’ seems to be a more plausible explanation. Learned non-use is a phenomenon associated with damage to the nervous system, in which the initial inability to perform movements with the paretic limb in the acute phase, and subsequent slow recovery at the neural level, result in difficulties in paretic limb motor performance, leading to a conditioned suppression of the use of the paretic limb [59, 60]. The notion of learned non-use implies the existence of latent, residual capacity of the paretic limb, which can be reduced by intensive, targeted training of the paretic limb [60, 61]. The improvements in paretic propulsion that we observed following task-specific gait training are in line with this notion.

As not every stroke survivor may have such latent residual paretic capacity [62], it would be of interest to identify—prior to the intervention—which individuals do and may thus benefit most. We indeed found that participants with greater propulsive asymmetry at baseline showed larger treatments gains in propulsion symmetry. We also tested whether the baseline propulsion capacity score was associated with post-intervention gains in propulsion, but we could not confirm this previously reported relationship [39]. Nevertheless, both these potential determinants can only be tested in a gait laboratory, which may not be practical for clinical implementation. As identifying those individuals who may benefit most from training may help improve individually-tailored rehabilitation, future research should focus on establishing simple baseline measures as reliable indicators of residual propulsive capacity of the paretic leg.

In addition to gains in paretic propulsion, we also expected to find training-induced improvements in peak paretic knee flexion in those participants with reduced knee flexion at baseline, as it is known that propulsion generation provides mechanical energy to flex the leg during swing [63, 64]. Although the difference just failed to reach statistical significance, it should be noted that following training peak knee flexion of the paretic leg had increased by almost 10° and, thereby, exceeded the minimal detectable change for peak knee flexion (i.e. 5.7° [65]) and the minimal clinically important difference for knee sagittal range of motion in stroke survivors (i.e. 8.48° [66]). As improved post-stroke knee kinematics may promote safe foot clearance [12], it might be interesting for future studies to investigate intervention effects on both propulsion and knee kinematics in a larger group of stroke survivors with reduced knee flexion during swing at baseline.

Beneficial effects of training were not only observed in gait kinematics and kinetics, but also pertained to performance of functional gait tasks and, importantly, daily-life walking activity levels. Maintaining sufficient levels of physical activity in daily life is of vital importance for stroke survivors, as it is one of the cornerstones of cardiovascular risk management [67]. Although following our intervention the total time of walking remained constant, the intensity of walking had significantly increased. The intensity of walking, measured by accelerometer counts, is known to increase with faster gait speed [68–70]. Our findings thus indicate that the increase in gait speed, as measured in our laboratory, also translated to walking in daily life.

A limitation of the current proof-of-concept study is that we did not include a control group. Yet, by conducting two baseline assessments (separated in time by five weeks), we were able to increase the likelihood that improvements in propulsion symmetry were indeed attributable to the intervention period. Another study limitation is the relatively small range of lower extremity motor impairments in our group of participants, thereby limiting the generalizability of the current findings to the stroke population at large. People with more severe post-stroke motor impairments often have profound propulsion asymmetry, but it is conceivable that they also have limited residual propulsion capacity. Indeed, a previous study found that these individuals experienced lower gains in outcome after intervention [32]. Our finding that greater propulsion asymmetry at baseline was associated with larger intervention effects in propulsion symmetry may, therefore, not be generalized to stroke survivors with more severe motor impairments.

## Conclusion

The finding that propulsion symmetry, gait speed, performance on functional gait tasks, and daily-life walking intensity had improved following task-specific training and persisted at follow-up hold promise for gait rehabilitation in individuals in the chronic phase after stroke. Future work should focus on identifying individuals with a latent propulsive capacity using simple measures at baseline, and confirm benefits of the used training concepts, in gait training settings with or without the use of an expensive robotic gait trainer, in a randomized controlled trial.

## Supplementary Information

**Additional file 1. Table S1.** Test statistics - Mixed Model Analysis.

## Data Availability

The data sets used during the current study are available from the corresponding author on reasonable request.

## Abbreviations

6MWT: 6-Minute Walk Test
FAC: Functional Ambulatory Categories
FGA: Functional Gait Assessment
HADS: Hospital Anxiety and Depression Score
ICF: International Classification of Functioning, disability and health
MAS: Modified Ashworth Scale
MMSE: Mini-Mental State Examination
MRC: Medical Research Council
SIS: Stroke Impact Scale
