# Supplementary material for: Task-specific training for improving propulsion symmetry and gait speed in people in the chronic phase after stroke: a proof-of-concept study
Source: J Neuroeng Rehabil. 2021 Apr 23;18:69. doi: 10.1186/s12984-021-00858-8 (PMC8062933; doi:10.1186/s12984-021-00858-8)
Supplement: Supplementary file 1 — Additional file 1. Table S1. Test statistics - Mixed Model Analysis. [file 12984_2021_858_MOESM1_ESM.docx]

| Additional table 1. Results of the mixed model analyses | | | |
| --- | --- | --- | --- |
|  | Baseline (average T0 and T1) vs. post-intervention (average T2 and T3) | Interaction T0 * post-intervention (average T2 and T3) | T2 vs. T3 |
| ***Propulsion measures*** |  |  |  |
| Propulsive impulse |  |  |  |
| Symmetry | F(79.392)=36.032 p<0.001 | F(79.499)=18.199 p<0.001 | F(77.489)=3.083 p=0.083 |
| Paretic leg | F(103.511)=4.730; p=0.032 | F(102.641)=1.794; p=0.183 | F(75.092)=0.010; p=0.922 |
| Non-paretic leg | F(103.629)=1.738 p=0.190 | F(103.817)=0.733 p=0.394 | F(75.780)=0.125 p=0.724 |
| Trailing limb angle – paretic leg | F(92.916)=26.025 p<0.001 | F(94.000)=23.347 p<0.001 | F(79.246)=1.129 p=0.291 |
| Ankle plantarflexion moment – paretic leg | F(103.739)=10.173 p=0.002 | F(103.291)=7.271 p=0.008 | F(77.532)=0.379 p=0.540 |
| ***Capacity measures*** |  |  |  |
| Gait speed | F(97.658)=19.430 p<0.001 | F(97.875)=14.402 p<0.001 | F(81.464)=3.954 p=0.050 |
| 6MWT | F(103.389)=8.671 p=0.004 | F(103.903)=2.122 p=0.148 | F(81.368)=0.306 p=0.582 |
| FGA | F(100.612)=5.694 p=0.019 | F(100.888)=1.823 p=0.180 | F(77.617)=1.870 p=0.175 |
| ***Mobility at home*** |  |  |  |
| SIS – Mobility | F(99.999)=2.091 p=0.151 | F(99.773)=3.682 p=0.058 | F(62.619)=2.464 p=0.122 |
| Activ8 walking |  |  |  |
| Total time | F(99.794)=2.636 p=0.108 | F(100.958)=0.794 p=0.375 | F(81.263)=0.092 p=0.762 |
| Total intensity | F(98.325)=9.142 p=0.003 | F(98.899)=3.752 p=0.056 | F(82.304)=0.468 p=0.496 |
| 6MWT: 6-Minute Walk Test; FGA: Functional Gait Assessment; SIS: Stroke Impact Scale. | | | |
